# Drug-Induced Liver Injury: An Institutional Case Series and Review of Literature

**DOI:** 10.1177/2324709618761754

**Published:** 2018-03-14

**Authors:** Vijay Gayam, Mazin Khalid, Binav Shrestha, Muhammad Rajib Hossain, Sumit Dahal, Pavani Garlapati, Arshpal Gill, Amrendra Kumar Mandal, Ruby Sangha

**Affiliations:** 1 Interfaith Medical Center, Brooklyn, NY, USA

**Keywords:** drug-induced liver injury, liver, failure, amiodarone, fluconazole, valproic acid

## Abstract

Drug-induced liver injury (DILI) is the most common cause of acute liver failure in the USA. DILI can be broadly classified as Intrinsic and Idiosyncratic. Identifying predictors and at-risk patients are challenging but can have a substantial clinical implication. This case report series demonstrates the importance of valproic acid, fluconazole, and amiodarone as potential hepatoxic agents of drug-induced liver injury leading to acute hepatic failure. The causality in all cases was established by Roussel Uclaf Causality Assessment Method/Council for International Organizations of Medical Sciences score and Naranjo Algorithm. Obesity, hypo-perfusion state, and concurrent hepatotoxic agent might identify at-risk patients. Further studies are required to understand the risk factors.

## Background

Drug-induced liver injury (DILI) is defined as a liver injury or disease due to medications, herbs, or other toxic substances. It is the most common cause of acute liver failure in the United States.^1^ DILI entails 2 broad categories, intrinsic (drugs that can cause liver injury in high doses) and idiosyncratic (unusual reaction of drugs in susceptible individuals).^2^ The diagnosis of DILI can be a clinical challenge as it may imitate any form of liver disease. Many studies have attempted to characterize the risk factors that culminate in fulminant hepatic failure.^3-6^ Identification of at-risk patients for a potential DILI may be beneficial, as early intervention may improve patient outcomes.^7^ The advent of genomic polymorphisms regarding drug metabolism in addition to improved biomarkers of DILI may aid in identifying cases of DILI.^7-9^

## Aim

We present 3 patients with acute fulminant hepatic failure from our hospital. We try to identify the risk factors and disease pattern in a series of fulminant hepatic failure cases. We aim to establish if any predictive features exist that can indicate at-risk patients. This case report series also demonstrates the importance of valproic acid, fluconazole, and amiodarone as potential hepatotoxic agents of DILI leading to acute hepatic failure.

## Methods

In this case series report, 3 patients with acute liver injury are presented. All eligible subjects included in the study met the criteria for the definition of DILI. The causality in all cases was established by Roussel Uclaf Causality Assessment Method/Council for International Organizations of Medical Sciences score and Naranjo Algorithm. All clinical and laboratory data were reconciled with the source documents before the causality assessment.

## Case Series

### Patient 1

A 45-year-old African American male presented to emergency department with complaints of fever and a pruritic inguinal rash. The patient had noticed a reddish-brown rash in the inguinal region for the past 4 days that was pruritic and malodorous. He denied any cough, chest pain, dyspnea, palpitations, dysuria, diarrhea, nausea, or vomiting. He had a past medical history of hypertension, diabetes mellitus, dyslipidemia, bilateral stage 3 gluteal decubitus ulcer, and paraplegia after a gunshot wound to the spine 26 years ago. He had a history of tobacco and alcohol abuse. He had no history of a chronic liver disease. The patient also reported a previous history of mild asymptomatic elevations in liver function tests (LFTs) during a 1-week course of oral fluconazole for candiduria, after which the drug was discontinued with no further complaints. On admission, he was febrile with a temperature of 102.4°F, a pulse of 117 beats per minute, and blood pressure of 170/106 mm Hg. Physical examination revealed an elevated, erythematous, and tender rash in the inguinal area with marks of skin excoriations consistent with pruritus. There was a bilateral stage 3 sacral decubitus ulcer visible with no signs of infection. There were no signs of asterixis, spider angioma, or organomegaly. His initial serum electrolytes, LFTs, comprehensive metabolic panel, and coagulation panel were all within the reference range. Hematological investigations showed a leukocytosis of 21 000 white blood cells/µL (reference 4500-11 000) with normochromic, normocytic anemia. A diagnosis of sepsis was made, likely due to inguinal candidiasis with superimposed bacterial cellulitis. The patient was started on intravenous (IV) normal saline, IV fluconazole 200 mg daily, and IV clindamycin. Blood, urine, and sputum cultures returned negative, and the patient had no further febrile episodes. However, on the third day, he developed acute liver failure (ALF) with an aspartate aminotransferase (AST) of 25 000 IU/L (reference 8-46 IU/L), an alanine aminotransferase (ALT) of 6500 IU/L (reference 7-55 IU/L), a γ-glutamyl transferase of 210 IU/L (reference 0-65 IU/L), an alkaline phosphatase (ALP) of 130 IU/L (reference 45-115 IU/L), a total bilirubin of 2.3 mg/dL (reference 0.1-1.2 mg/dL), and a direct bilirubin 0.4 mg/dL (reference <0.3 mg/dL). Coagulation studies noted an international normalized ratio (INR) of 3.2 (reference 0.8-1.1), a prothrombin time of 25 seconds (reference 11-14 seconds), and a partial thromboplastin time of 28 seconds (reference 25-35 seconds). Hepatitis panel, Epstein-Barr virus, cytomegalovirus, and human immunodeficiency virus tests were negative. An abdominal ultrasound showed mild hepatomegaly. Considering the temporal association combined with no other probable etiologies for the patient’s worsening ALF, drug-induced hepatotoxicity secondary to fluconazole was suspected and fluconazole was stopped. Three days after discontinuation of the drug, the LFTs and coagulation studies improved. The patient’s prompt clinical recovery after withholding the drug corroborated our diagnosis of fluconazole-mediated ALF. Clindamycin was continued during this time, and causation was ruled out as its continuation did not result in any incident. Also, the patient had been previously treated with clindamycin without any event.

#### Causality Assessment

The causality of drug-induced hepatotoxicity, in this case, was established by Roussel Uclaf Causality Assessment Method/Council for International Organizations of Medical Sciences score as follows:

Hepatocellular, second exposure, onset of <5 days (+1), time from withdrawal of drug until reaction onset <15 days (1), risk factors being alcohol (+1), age <55 (0), >50% improvement in 8 days (+3), no concomitant therapy (0), excluded non–drug-related causes: rule out (+2), response to readministration positive (+3) with total score of 11 indicating “Highly Probable” (>8).

This drug-related adverse event was confirmed by Naranjo Algorithm as follows:

Previous reports positive (+1), adverse events appeared after the suspected drug was given (+2), with the transaminases improving after the discontinuation of the drug (+1), and the adverse reaction appearing after the readministration of the drug (+2), with no alternative causes to explain this adverse reaction (+2), placebo not been given (0), drug levels not done (0), without changing the administered dose of the drug (0), similar reaction in the past with the same drug (+1), and the adverse event confirmed by objective evidence (+1) with a total score of 10 which is >9, thus “Definite ADR.”

The temporal association also strongly proves drug-induced hepatotoxicity in this case.

### Patient 2

A 56-year-old African American male was referred to the emergency department from his dialysis center following an episode of tachycardia. Past medical history was notable for end-stage renal disease, hypertension, coronary artery disease, seizure disorder, and atrial fibrillation. The patient stated having fatigue for the past month but denied any other complaints including fever, abdominal pain, nausea, vomiting, diarrhea, or constipation. On admission, his pulse was irregular at 150 beats per minute, and his blood pressure was 136/95 mm Hg. The patient was not in any apparent distress and was oriented to person, place, and time. His physical examination was remarkable only for decreased breath sounds and rales in the right lower lobe of the lung along with bilateral lower extremity edema. There were no signs of icterus, palmar erythema, spider angioma, abdominal ascites, tenderness, and organomegaly. Electrocardiogram revealed atrial flutter and was subsequently started on an IV diltiazem drip. However, he continued to have a persistent atrial flutter, which was not rate-controlled and was started on amiodarone infusion at the rate of 28.8 mg per hour. The echocardiogram was done, and showed severe systolic heart failure with an ejection fraction of 25%. Shortly after the initiation of amiodarone, the patient’s LFTs started to deteriorate with worsening coagulopathy and aminotransferases rising to 65 to 70 times the upper limit of normal (ULN). Peak levels of AST, ALT, and the INR were 3000 IU/L, 1500 IU/L, and 24, respectively. Amiodarone along with his antiseizure medications, phenobarbitone and phenytoin, were discontinued as the patient was seizure free for more than 2 years. Ultrasound of the abdomen showed hepatomegaly with diffusely increased echogenicity. The patient had no urticaria, rashes, or eosinophilia. There was no significant hypotension. The retrospective review did not reveal the use of alcohol or any other hepatotoxic drug beside phenytoin and phenobarbitone for seizures. Viral hepatitis panel, cytomegalovirus, Epstein-Barr virus, herpes simplex virus, human immunodeficiency virus, as well as blood cultures and vasculitis workup were all negative. Despite the discontinuation of the amiodarone, the LFTs continued to deteriorate, and he developed multiple organ dysfunctions with eventual cardiac arrest and death. A diagnosis of acute fulminant liver failure secondary to amiodarone-induced shock liver was established.

#### Causality Assessment

The causality of drug-induced hepatotoxicity was established by Roussel Uclaf Causality Assessment Method/Council for International Organizations of Medical Sciences score as follows:

Hepatocellular, first exposure, onset of <5 days(+1), time from withdrawal of drug until reaction onset <15 days (0), risk factors being alcohol (0), age >55 (+1), course worsened (−1), no concomitant therapy (0), excluded non–drug-related causes: ruled out (+2), previous information on hepatotoxicity—reaction labeled in the product characteristics (+2), response to readministration not available (0) with total score of 5 indicating “Possible ADR.”

The adverse drug reaction (ADR) was confirmed by Naranjo Algorithm as follows:

Previous reports positive (+1), adverse events appeared after the suspected drug was given (+2), with the transaminases not improving after the discontinuation of the drug (0), and the drug was not readministered (0), with no alternative causes to explain this adverse reaction (+2), placebo not been given (0), drug levels not done (0), without changing the administered dose of the drug (0), no similar reaction in the past with the same drug (0), and the adverse event confirmed by objective evidence (+1) with a total score of 6 which is <9, thus “probable ADR.”

Additionally, the temporal association is strongly suggestive of drug-induced hepatotoxicity.

### Patient 3

A 27-year-old African American male presented to the emergency department with a 3-day history of hypersomnolence, fatigue, and anorexia. The patient denied fever, chills, sweating, headaches, abdominal pain, nausea, or vomiting. He had a past medical history of asthma, chronic marijuana and tobacco use, bipolar disorder, schizoaffective disorder, and multiple suicidal attempts. His medications included haloperidol, benztropine, aripiprazole, and was recently started on valproic acid (VPA). On admission, he had a temperature of 98.2°F, blood pressure of 117/82 mm Hg, heart rate of 70 beats per minute, and a respiratory rate of 20 beats per minute. Physical examination revealed a somnolent patient with an ataxic gait, scleral icterus, and prominent asterixis. There was no appreciable lymphadenopathy or organomegaly, and no signs of palmar erythema, gynecomastia, shifting dullness, or spider angioma. Initial laboratory results were remarkable for an AST level of 12 000 IU/L (reference 8-46 IU/L), ALT of 7000 IU/L (reference 7-55 IU/L), an ALP of 96 IU/L (reference 45-115 IU/L), total bilirubin of 1.5 mg/dL (reference 0.1-1.2 mg/dL), and serum ammonia of 184 µmol/L (reference 15-45 µmol/L). The patient also had thrombocytopenia and coagulopathy. Abdominal ultrasound and computed tomography of the head were both unremarkable. Viral hepatitis serology and acetaminophen toxicity were both negative. Urine toxicology was positive for ethyl alcohol at 9 mg/dL. An ADR to VPA was suspected, and VPA was discontinued with the initiation of lactulose for hyperammonemia. On the second day after discontinuing VPA, his LFTs started to trend downward and his clinical symptomology of hyperammonemia improved. By the eighth day, his ALT and AST levels had dropped to 634 IU/L and 61 IU/L, respectively, and his total bilirubin was within reference range at 0.8 mg/dL. A final diagnosis of VPA-induced DILI resulting in hepatic encephalopathy was made.

#### Causality Assessment

The causality of drug-induced hepatotoxicity in this case was established by Roussel Uclaf Causality Assessment Method/Council for International Organizations of Medical Sciences score as follows:

Hepatocellular, second exposure, onset of <5 days(+1), time from withdrawal of drug until reaction onset <15 days (+1), risk factors being alcohol (+1), age <55 (0), >50% improvement in 8 days (+3), no concomitant therapy (0), excluded non–drug-related causes: rule out (+2), response to readministration positive (+3) with total score of 11 indicating “Highly Probable” (>8).

This drug-related adverse event was confirmed by Naranjo Algorithm as follows:

Previous reports positive (+1), adverse events appeared after the suspected drug was given (+2), with the transaminases improving after the discontinuation of the drug (+1), and the adverse reaction appearing after the readministration of the drug (+2), with no alternative causes to explain this adverse reaction (+2), placebo not been given (0), drug levels not done (0), without changing the administered dose of the drug (0), similar reaction in the past with the same drug (+1), and the adverse event confirmed by objective evidence (+1) with a total score of 10 which is >9, thus “Definite ADR.”

The temporal association also strongly proves drug-induced hepatotoxicity in this case.

## Discussion

The DILI is characterized as any form of liver injury from either prescription drugs or over-the-counter medications. Two types of drug hepatotoxicity are described in literature—idiosyncratic (unpredictable) and nonidiosyncratic (predictable).^10,11^ DILI can be further classified into acute, chronic, cholestatic, hepatic, or mixed, based on the abnormalities of liver function tests.^12^ The mechanism of DILI may be immune-mediated or metabolic due to direct toxicity from the administered drug.^13-15^ DILI that is idiosyncratic accounts for 11% of the cases of ALF in the United States, and it is the most common cause of ALF.^4,16^ One review of the literature demonstrated that approximately 20 new cases of DILI per 10 000 persons occur each year.^17,18^ An Icelandic study conducted by Björnsson showed the incidence of idiosyncratic DILI in a population-based cohort. The study identified 96 cases of DILI over the span of 1 year from 2010 to 2011, with the crude annual incidence rate being 19.1 (95% confidence interval = 1.54-23.3) cases per 10 000 inhabitants.^18^ There are no established risk factors for DILI, but factors such as hepatic metabolism, drug lipophilicity, dosing, preexisting liver injury, and genetic predispositions to specific drugs have been attributed to DILI.^19^

**Figure 1.**
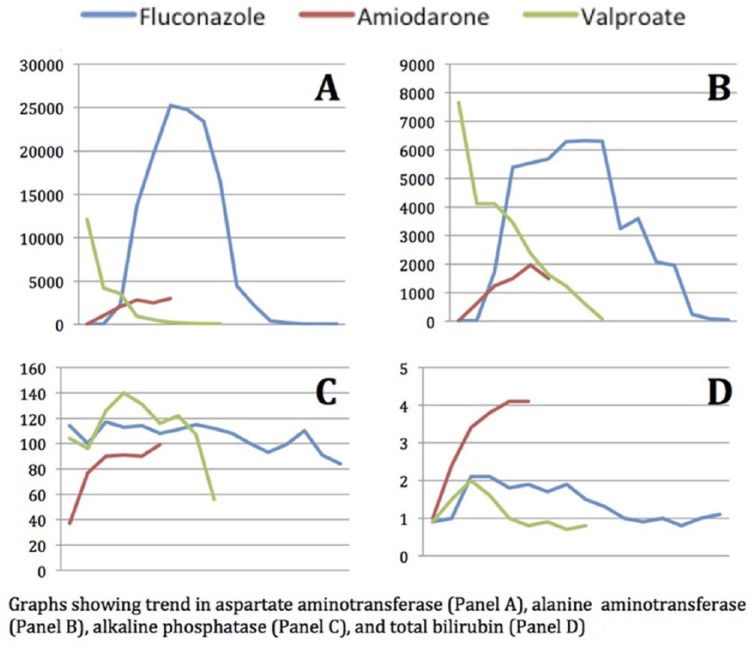
Trends in liver function tests.

The mechanism of DILI remains unclear, but there are proposed models in the literature. Russman et al describe a 3-step model of DILI, in which mitochondrial stress alters mitochondrial permeability leading to apoptosis and necrosis.^20^ The model may help explain the mechanism of hepatocellular hepatotoxicity, which occurred in each of our patients, as proven by the *R* score—a ratio of ALT/ULN to ALP/ULNALT—of greater than 5. The onset of liver injury in this case series varied from 1 day to several days. Onset and offset of hepatotoxicity were unrelated to the half-life or duration of action of the offending medication and lends support for the idiosyncratic nature of DILI in our patients. In all the cases, features of hypersensitivity such as fever, rash, eosinophilia, and autoimmunity were not evident. Despite this, the lack of hypersensitivity features in our case series does not entirely exclude an immunological mechanism. There were wide variations in the peak values of biochemical markers, which are unlikely to contain a prognostic value (Figure 1). The higher level of peak AST was seen than peak ALT. However, the peak level of liver enzymes did not correlate to prognosis. Interestingly, on close observation, the resurgence of AST after the offset had begun was an indicator of mortality. Whether this finding is clinically relevant remains unknown. In the cases of recovery, the total bilirubin never rose beyond 2.5 mg/dL, while in the case of mortality, the bilirubin rose to beyond 3 mg/dL. Initially, the AST/ALT ratio is >1, and as the offset of ALT lags after AST, over time ALT was seen greater than AST, thus demonstrating an inverse of the ratio of AST to ALT <1. This reversal of AST/ALT ratio is an indicator of survival and recovery in our patients. The last marker to normalize was ALP. As a result, resolution in our cases would be indicated by normalization of all LFTs, specifically ALP in our patients.

Review of the literature suggests many case reports of DILI involving a wide array of drugs.^21^ The most commonly implicated drugs are acetaminophen, nonsteroidal anti-inflammatory drugs, isoniazid, and amoxicillin/clavulanate.^22^ Amiodarone is known to cause hepatotoxicity in both oral and IV forms.^23-26^ The etiology is not clearly identified; however, the oral form is thought to cause hepatotoxicity due to direct mitochondrial damage caused by its accumulation in mitochondria. On the other hand, the IV form is mixed with a chemical (polysorbate), which is attributed to the increase incidence of hepatotoxicity with the IV form when compared with the oral form.^27-28^ Hepatitis E also remains an alternative diagnosis in patients in whom DILI is suspected and should be ruled out.^29^ The primary challenge is to establish a causative relationship between a particular medication and liver injury. Several clinical scoring scales have been developed to assist in finding these associations. The most common criteria set used for the diagnosis of DILI is the Rousse Uclaf Causality Assessment Method of the Council of International Organization of Medical Science (RUCAM/CIOMS).^30,31^ This scale scores several risk factors such as pregnancy, consumption of alcohol, and age while categorizing the form of DILI into the 3 patterns of hepatocellular, cholestatic, and mixed.^31^ The limitation of this scale as well as others is that in clinical practice these criteria sets are not regularly used for the diagnosis of DILI. The Drug-Induced Liver Injury Network sponsored by the National Institutes of Health (Bethesda, MD is a research consortium in place to acquire improved data regarding drug hepatotoxicity and to provide access to a case registry.^32,33^

Diagnosing DILI is difficult as there are no specific biomarkers or histologic features that identify a drug as the cause of hepatic injury.^2^ There have been suggestions to use biomarkers in high-risk patients, including microRNA 122, which is a specific marker of DILI.^1,32^ In our cases, biomarkers were not used; instead, the causality was objectively defined through the RUCAM/CIOMS score and Naranjo algorithm. Vital components to attributing a drug to liver injury include a prior history of drug exposure, discontinuation of the drug leading to improvement in liver injury, the recurrence that is severe and rapid on rechallenge of a drug, and exposure to a drug that has a history of causing DILI in other patients. Once the diagnosis was confirmed to be DILI in our patients, the management was supportive, as currently there is no definitive antidote for idiosyncratic DILI.^2^ Recovery and prognosis involve discontinuation of the offending agent, which often results in clinical improvement. The decision to discontinue an offending agent is based on liver enzyme values. One study suggests discontinuing administration of the drug when ALT is more than 5 times the ULN for 3 weeks, ALT is more than 8 times the ULN, ALT is more than 3 times the ULN with serum bilirubin also more than 2 times the ULN, prothrombin time/INR is more than 1.5 times the ULN or in the presence of symptoms suggesting liver injury.^30^ Specific treatments are not indicated except L-carnitine in cases of DILI caused by VPA and N-acetylcysteine for cases caused by acetaminophen, respectively.^34,35^

All 3 cases in our series were male. This is a contrast to the literature showing a female predominance in DILI.^21^ Each of the 3 patients was obese with a body mass index >30 kg/m^2^. Two out of the 3 cases also involved the use of other potentially hepatotoxic agents, including alcohol. Similarly, 2 of our cases occurred during a hypoperfusion state in the patient. Another important issue to note was the worse outcomes associated with reexposure to a medication known to cause prior liver impairment in the patient (Table 1).

**Table 1.** Characteristics of the Patients.

	Case 1	Case 2	Case 3
Implicated drug (route)	Fluconazole (intravenous)	Amiodarone (intravenous)	Valproate (oral)
Age (years)	45	53	27
Sex	Male	Male	Male
Body mass index (kg/m^2^)	31.9	39.2	42
Past medical history	Hypertension, diabetes mellitus, dyslipidemia, paraplegia due to gunshot injury, stage 3 decubitus	Hypertension, chronic kidney disease on dialysis, seizures, atrial fibrillation	Chronic obstructive pulmonary disease
Life style habits	Smoker, alcoholic	Former smoker	Alcoholic, smoker
Significant drug history	History of fluconazole-induced asymptomatic altered liver function tests	On phenytoin and phenobarbital	Recently started on valproate
Clinical presentation and diagnosis	Sepsis due to cellulitis	Atrial flutter	Drowsiness due to hyperammonic encephalopathy
Complete blood count at presentation			
Hemoglobin (g/dL)	13.5	6.2	14.8
White blood cell count (per µL)	21 000	2600	5800
Platelet count (per µL)	260 000	133 000	87 000
Basic metabolic panel			
Sodium (mmol/L)	136	135	134
Potassium (mmol/L)	4	4.3	4.0
Chloride (mmol/L)	99	97	101
Bicarbonate (mmol/L)	27	28	21
Blood urea nitrogen (mg/dL)	12	66	20
Creatinine (mg/dL)	1.6	9.2	1.1
Outcome	Recovery	Death	Recovery

Recent advances in proteomics, toxic genomics, and microRNA can improve the identification of both risk factors and underlying hepatotoxicity in DILI. The association between human leukocyte antigen (HLA) class II and its predisposition to the antibiotic amoxicillin-clavulanic acid in relationship to DILI were confirmed via a genome-wide association study.^36^ There is ongoing research on the protective effect of HLA-DRB*07 allele family, as well as the use of microRNA as a potential marker of DILI.^13,36^ Keratin variants and their use as a predictive outcome of ALF along with the use of N-acetylcysteine as a protective mechanism against DILI with the concurrent use of antituberculosis medications are also being studied.^13,36,37^ This ongoing research in the genetics of predisposition and pathophysiology of DILI can aid in not only predicting but also preventing DILI in clinical practice.^36^

## Conclusion

Though highly variable regarding patient characteristics, presentations, and outcomes, all our patients with DILI were obese males and likely to be having some hypoperfusion state or taking a concurrent hepatotoxic agent. Ongoing and future studies to predict the risks and outcomes of DILI are needed.
